# Individual differences in decision-making: evidence for the scarcity hypothesis from the English Longitudinal Study of Ageing

**DOI:** 10.1098/rsos.220102

**Published:** 2022-10-26

**Authors:** Richard J. Tunney, Richard J. E. James

**Affiliations:** ^1^ School of Psychology, Aston University, Birmingham, UK; ^2^ School of Psychology, University of Nottingham, Nottingham, UK

**Keywords:** impulsivity, socio-economic classification, indices of multiple deprivation, scarcity

## Abstract

We report the results of a pre-registered analysis of data from the English Longitudinal Study of Ageing that was designed to test the hypothesis that economic scarcity is associated with individual differences in decision-making. We tested this hypothesis by comparing time preferences for different socio-economic groups and in geographical areas ranging from the most deprived to the least deprived in England using the English indices of multiple deprivation. The data supported this hypothesis: people in the most deprived areas were more likely to prefer smaller-sooner rewards than people from the least deprived areas. Similarly, people in technical or routine occupations tended to prefer smaller-sooner rewards than people in professional or intermediate occupations. In addition, we found that gender, cognitive function and subjective social status also predicted time preferences. We discuss these results in the context of theoretical models of scarcity-based models of choice behaviour and decision-making.

## 1. Introduction

Why are some people more impulsive than others? The question is important because impulsivity is a risk factor for many of the behaviours that society considers problematic, either when they are prohibited such as drug abuse, or are permitted but can be taken to excess such as gambling, smoking tobacco, or drinking alcohol. Impulsivity also features as a criterion in a wide range of other psychiatric disorders, for example, antisocial Personality Disorder, bipolar disorder and attention deficit hyperactivity disorder. It is important, therefore, to understand why some people are more prone to making impulsive decisions. Evidence suggests that impulsivity, and symptoms of psychiatric disorders that feature impulsivity as a criterion, may not be normally distributed across the population [1–3]. This suggests that differences in environmental or personal circumstances might give rise to differences in levels of impulsivity. A reasonable question is to ask what these circumstances might be. In this paper, we explore the possibility that individual differences in impulsivity arise from differences in economic environments. We conducted a secondary analysis of the English Longitudinal Study of Ageing to test the scarcity hypothesis of impulsivity by comparing impulsivity in different socio-economic groups and in areas that differ in levels of deprivation.

### 1.1. The scarcity hypothesis

Resource insecurity in animals, and its analogue, economic scarcity in humans, is a leading candidate of an environmental driver of choice behaviour. There are a number of plausible explanations why scarcity or resource insecurity might affect decision-making. In some models, scarcity directly affects decision-making because a scarcity of resources makes any decision about money more salient, more frequent and more imminent than for more well-off people [4,5]. People in lower income brackets tend to spend a greater proportion of their income on housing, and are less likely to own their own homes. Similarly, people from less affluent socio-economic groups are less able to deposit money in savings accounts, and are more likely to use any disposable income on short-term outgoings [6,7], spend a higher proportion of their income on lottery tickets [8], and are willing to accept higher rates of interest on loans even when eligibility is held constant [9]. Experimental manipulations designed to induce a ‘poverty mindset' generally support this scarcity hypothesis. In a series of experiments reported by Shah *et al.* [4], participants were given either small or large budgets to use in a series of economic games. When their budgets ran out, loans were made available to continue playing. The participants given smaller budgets were more likely to borrow, and did so at higher rates of interest than the participants given larger budgets.

Alternatively, it has been claimed that some individual differences in human traits resemble differences between cultures and even species. Life-history theory [10–13] suggests that behaviour reflects an adaptation or sensitization to early environments (life history) in which behavioural strategies adapt to become relatively fast or relatively slow depending on environmental circumstances or ecological niche. As life expectancy decreases, individuals will respond by reproducing at an earlier age and have a higher reproductive rate. The evidence for this model is frequently based on cross-species comparisons which may not be compelling when generalized to human decision-making [14]. However, there is some evidence that countries with lower incomes (GDP) tend to have higher mortality rates, and higher reproduction rates than countries with higher incomes [15]. There is considerable evidence that income is related to time preferences [16–22]. Evidence from the UK Millennium Cohort Study suggests that women in more deprived areas have their first child at a younger age, and that their reproductive rates are higher [23]. The reproductive adaptations have been associated by some researchers with differences in individual traits including pro-sociality, risk- and time-preferences. However, despite considerable theoretical work [24,25] and a recent meta-analysis [26,27], to our knowledge there have been no direct tests of trait impulsivity in the form of time preferences and objective measures of social status or relative deprivation. Although life-history theory gives a general description of what reproductive strategies are most adaptive to different environmental conditions, it does not in itself explain the mechanism by which early life experience would affect the cognitive processes involved in decision-making.

Decision-making in humans shares many similarities with foraging behaviour in other animals [28], and choice tasks designed to measure impulsivity are often strikingly similar to models of foraging [29–31]. The foraging behaviour of groups and the choice behaviour of individuals are described by synonymous mathematical models [30,32]. Individual differences in decision-making may result from the foraging responses that make sense in different environmental conditions such as scarcity of resources. The optimal forager should titrate their behaviour to the availability of resources. For example, in periods of abundant resources it may not make sense to deplete the available resources since these could be preserved for the future. Thus, in periods of abundance it is more optimal to exhibit self-control or delay gratification. However, in periods of scarcity it makes more sense to deplete the available resources even if that results in an uncertain future. For example, obesity is associated with perceived or actual food insecurity [33,34], potentially as an insurance against future deprivation [35]. This could be both metabolic in the sense that people and other animals tend to lay down greater fat stores and seek calorific foods when they encounter unpredictable food resources; and social in the sense that these effects appear to be greater in lower status than higher status individuals [34].

### 1.2. Impulsivity as time preference

Impulsivity is a multi-faceted construct, but it is time preferences in humans that are most similar to foraging models of animal choice behaviour. Time preference refers to the relative preference for smaller-sooner rewards or delayed gratification for larger-later rewards [36–39]. Delay discounting is a psychophysical measure of time preference in which people are given a series of choices between monetary rewards after increasing delay periods, for example from 1 day to 10 years [40]. The relative preferences for $100 tomorrow rather than $1000 in, say, 10 years time indicates the degree to which the individual discounts the future value of the $1000. By titrating the delay period, it is possible to derive a single parameter estimate (*k*) of an individual's or a group's relative preference for smaller-sooner rewards over larger-later rewards. The validity of time preferences as a measure of trait impulsivity is indicated by its close predictive relationship with impulsive behaviours associated with addiction. Steeper discount rates are associated with a range of addictive behaviours including tobacco smoking [41], severity of alcohol use disorders [42], gambling [43] and cocaine and heroin use [44]. Evidence is also emerging that steeper discounting is also associated with behaviours that are not at present formally regarded as behavioural addictions, but which may be categorized as such in the future, including eating disorders [45] and Internet gaming disorder [46].

The association between psychophysical and psychometric measures of impulsivity lends delay discounting a degree of construct validity. Discount rates are moderately correlated with established psychometric measures of impulsivity such as the Barratt impulsivity scale (BIS-11) [47–49] the UPPS impulsivity scale [50,51]. These psychometric measures, however, contain a range of factors that we might not expect to be related to time preferences, and frequently are only poorly related to each other.

### 1.3. Overview of the study

In the study that follows, we make use of secondary data to confirm whether impulsivity in the form of time preference is unevenly uniformly distributed across socio-economic groups. In doing so, we consider different markers of economic scarcity. We used the indices of multiple deprivation (IMD) to test the scarcity hypothesis to examine whether deprivation predicts time preferences. The IMD incorporate different aspects of economic scarcity, such as lack of income, but also fewer opportunities to access education, housing and other opportunities. Thus, this does not just capture individual overall economic circumstances, but also the extent to which these environments lead to greater opportunity. We also modelled socio-economic status. Socio-economic status is typically recorded using occupation, and although occupations differ considerably in remuneration this does not imply poverty, and not every member of even the least affluent occupational groups experience significant and prolonged hardship. We also accounted for other common indices of socio-economic status, specifically years spent in education. Access to the IMD is restricted and requires a special licence from the UK Data Service. We pre-registered our method and analysis with the UK Data Service in order to obtain this licence and also with the Open Science Framework (https://osf.io/x2qyr/?view_only=7595cab8a1e84e9fbcea8f3ab17e68d1) prior to obtaining access to the data. Subsequent extended analyses were conducted to test whether any observed effects might be confounded by factors (e.g. subjective social status, cognitive function) that could explain these relationships.

## 2. Material and methods

The data were drawn from the English Longitudinal Study of Ageing (ELSA). This is a representative sample of about 11 000 people aged over 50 years living in England [52]. The first wave of the survey began in 2002, and subsequent waves occurred at 2-year intervals. The sample is periodically refreshed to compensate for attrition and to incorporate new cohorts of older adults. Wave 5 was conducted between July 2010 and June 2011. Of the 10 274 participants in Wave 5, 1557 respondents aged 50–74 were randomly pre-selected to complete an additional ‘risk module' that included a measure of time preference that is closely related to delay-reward discounting. Of these 1062 consented to completing the time preference measure. The remainder did not consent to this part of the survey. As a longitudinal survey, the data for each of the variables were collected or refreshed on each wave of the survey; however, all of our analyses were based on the data collected during Wave 5 when the risk module was administered. The average age of the 479 of the participants who identified as men was 63.65 years (s.d. = 5.938), the average age of the 583 participants who identified as female was 62.68 (s.d. = 6.09).

### 2.1. Time preference

The measure of time preference used in the ELSA survey was called the ‘rectangle game’. The game was a time preference task with 12 items in which participants were asked to choose between a fixed £25 in two weeks and [£26, £28, £30, £32, £35, £38] in one month; and between £25 in two weeks and [£26, £30, £35, £37, £40, £45] in two months. The choices were presented on a laptop computer one at a time in the order described here. We summed the number of large-later choices as our dependent variable. The resulting scores range from 0 = all preferences are smaller-sooner to 12 = all preferences are for larger-later rewards. Lower scores indicate greater impulsivity, and larger scores indicate lower impulsivity. As an incentive the participants were each paid £20 for taking part in the survey plus an endowment of £10 to play the two games. They were told that they could win an additional £70 depending on the choices that they make, or lose £5 from the endowment. The computer randomly selected one of the trials from the game as payment.

### 2.2. English indices of multiple deprivation

The English indices of multiple deprivation (IMD) [53] is the official UK government measure of relative deprivation in England. The IMD divides England into 32 844 neighbourhoods of around 650 households or 1500 residents. Wave 5 uses data from the 2010 IMD. The overall index is composed of income deprivation, employment deprivation, education, skills and training deprivation, health deprivation and Disability, crime, barriers to housing and services, and living environment deprivation (see electronic supplementary material, tables S1 and S2). Each domain includes a range of indictors of deprivation. For example, the income deprivation domain counts the number of people in each area in receipt of specific welfare support, while living environment includes measures such as air quality and traffic accidents (further details are provided by the Department for Communities and Local Government [53]). The scores from each domain are weighted before being summed. In our data, the range is from 0.53 to 87.80. For our purposes, the data are collapsed into quintiles of deprivation from 5 = most deprived to 1 = least deprived. We used these quintile scores as a categorical predictor variable.

### 2.3. Socio-economic status

Socio-economic status is recorded in the UK by occupation. The ELSA survey recorded socio-economic status using the national statistics socio-economic classification (NS-SEC). This system categorizes respondents' occupations into a number of categories. We used the five-group classification, in which 1 = managerial, administrative and professional occupations, 2 = intermediate occupations, 3 = small employers and own account workers, 4 = lower supervisory and technical occupations, 5 = semi-routine and routine occupations. These are intended to be nominal categories rather than ordinal classes [54] and so were dummy coded. The number of data points in each category are shown in electronic supplementary material, table S2. Although they do not directly measure economic scarcity, these are related to annual income and level of educational qualification.

### 2.4. Cognitive function

Cognitive function was assessed using a composite measure from five different tasks completed as part of the ELSA's cognitive function module, designed to test prospective memory, immediate recall, delayed recall, fluency and attention. This approach has been previously described by James & Ferguson [55].

At the very beginning of the module, participants were told that they would be given a clipboard and pencil during the cognitive function testing. They were also told that when they received these, they should write their initials in the top left-hand corner of the paper (designed to test prospective memory). Performance on this test was scored from 0 to 5 (5 = completed task correctly without prompting, 4 = partially completed the task (either wrote initials elsewhere or something in top left corner) without prompting, 3 = did something else, or declared they did not remember what to do without prompting, 2 = completed task after prompting, 1 = partially completed task after prompting, 0 = did nothing or failed to remember after prompting).

Participants were then randomly assigned to receive one of four different sets of words, which were then read aloud to the participant. The participant was then asked to tell the interviewer the words they could recall (immediate recall), and at the end of the module they were asked again without warning to recall the same list of words (delayed recall). During the module, participants were also instructed to say aloud as many animal names as they could think of in 60 s (fluency). Participants were also asked to complete a letter cancellation task (attention), in which they were instructed to cross out all instance of two letters on a sheet of text. Performance on this was measured in terms of the number of letters cancelled, minus the number of mistakes.

To develop the composite measure, these scores were entered into a principal components analysis. Parallel analysis indicated that all items loaded onto a single component (electronic supplementary material, table S3). As such, component scores were extracted using the regression method to index executive function.

### 2.5. Subjective social status

Respondents were given the McArthur scale of subjective social status [56]. This is a one-item scale, in which participants are presented with a ladder with 18 rungs. Participants are told that the top rung on the ladder represents the people with the best jobs, the most money and education, and that the bottom rung represents the people who are the worst off, with the least money, least education, and the worst or no jobs. Respondents were asked to indicate where they would place themselves on the ladder on a scale from 5 = worst off, to 100 = best off. Each rung of the ladder represented a 5-point increment.

### 2.6. Age education ended

We included the age that education ended as a predictor variable in our analyses because it is a good proximal indicator of relative deprivation and social status [57]. It is indicative of the qualifications that a person may or may not have obtained. In older adults such as those in this sample it tends not to be associated with intelligence because older adults may not have had the opportunities or expectations for education [58]. The average age that participants left education was 16 years (table 1). Three-hundred and eighty left school aged 15 or younger and would have therefore left school without any qualifications. Three-hundred and forty one left school at aged 16 or 17 and would have obtained school leaver certificates (O-Levels or equivalent). Two hundred and seventy-nine left education after age 18 and would have obtained higher qualifications.

**Table 1.** Descriptive statistics for continuous variables entered into the regression models. The number of larger-later choices was the dependent variable.

variable	mean	s.e.	min	max	*n*
larger-later choices	7.670	0.127	0	12	1063
age	63.120	0.185	50	75	1063
age education ended	16.603	0.067	14	19	745
cognitive function	−0.0008531	0.03068840	−5.69987	2.58287	1063
subjective social status	59.442	0.524	5.000	100.000	986

### 2.7. Statistical analysis

Ordinal least squared multiple regression models were estimated in both the pre-registered and extended analyses, with time preference as the dependent variable. For the pre-registered analysis, we entered age, gender (dummy coded with 1 = men, 0 = women), 5-factor NS-SEC, quintile indices of multiple deprivation (from 1 = least deprived to 5 = most deprived), and age education ended as predictor variables (table 1 and electronic supplementary material, table S2), and the number of larger-later choices as the dependent variable. We dummy coded the NS-SEC and used managerial as the reference category because we expected this group to be the least impulsive and because it was also the largest group (*n* = 394). We also dummy coded the indices of deprivation with the least deprived group as the reference category. In the extended analysis, we included cognitive function (standardized) and subjective social status. We omitted age education ended from this analysis, since it was not a significant predictor and necessitated the exclusion of 317 otherwise valid data points. We only included cases where there were valid data points for each of the variables. This led to there being a slightly larger sample size in the extended analysis. The survey coded six reasons for missing data: ‘refusal', ‘don't know', ‘error', ‘no valid answer', ‘not completed' or ‘not applicable'.

## 3. Results

The average number of larger-later choices was 7.670 (s.e. = 0.127). Descriptive statistics for each predictor variable are shown in table 1. Correlations between the variables are shown in table 2. We used Spearman's *ρ* to determine the association between socio-economic groups and the index of multiple deprivation. The two were moderately correlated but were not co-linear (*ρ* = 0.228, *p* < 0.001). The data met the necessary assumptions for multi-collinearity and homoscedasticity.

**Table 2.** Correlation coefficients for the continuous variables in the analyses.

	age	index of deprivation	cognitive function	subjective social status	age education ended
later	0.025	−0.146**	0.149**	0.165**	0.184**
age		−0.014	−0.289**	−0.020	−0.250**
index of deprivation			−0.144**	−0.204**	−0.273**
cognitive function				0.215**	0.381**
subjective social status					0.424**

**denotes significance at the 0.01 level.

### 3.1. Pre-registered analysis

**Figure 1.**
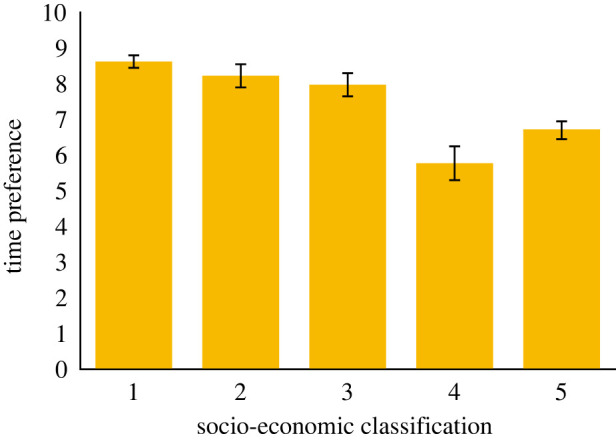
Showing time preferences (average number of larger-later choices) by socio-economic classification (1 = managerial, administrative, and professional occupations, 2 = intermediate occupations, 3 = small employers and own account workers, 4 = lower supervisory and technical occupations, 5 = semi-routine and routine occupations).

**Figure 2.**
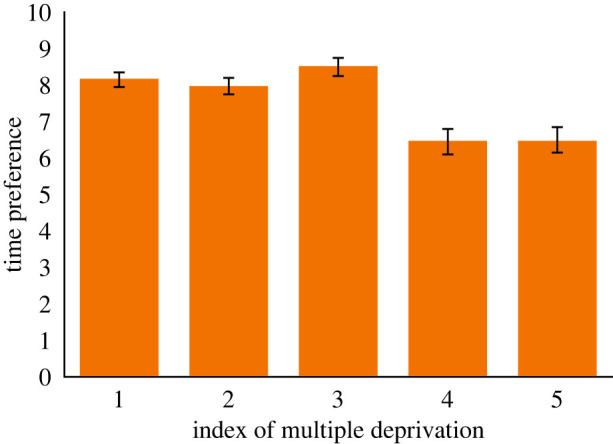
Showing time preferences (average number of larger-later choices) for each quintile index of multiple deprivation from 1 = least deprived to 5 = most deprived.

**Figure 3.**
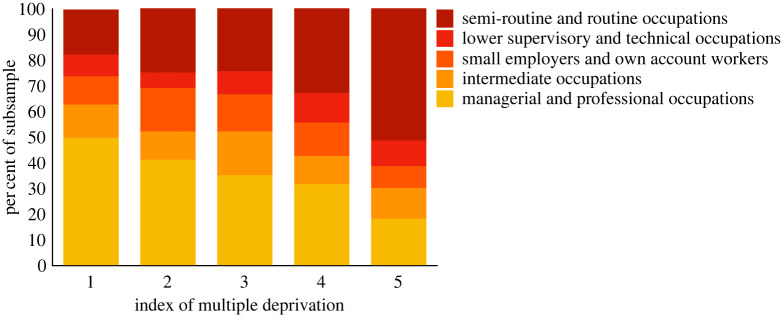
Showing the percentage of each occupational category in each quintile index of multiple deprivation from 1 = least deprived to 5 = most deprived. Nearly 50% of the sample in the least deprived areas were in managerial or professional occupations. Over 50% of people in the most deprived areas were in semi-routine and routine occupations. Percentages were computed excluding incomplete responses or responses recorded as ‘other'.

We began with our pre-registered analysis (*n* = 746). Figure 1 shows time preferences for each socio-economic group. Figure 2 shows time preferences for each index of multiple deprivation quintile. Figure 3 shows the percentage of people in each socio-economic classification in each IMD quintile. Nearly half of people in the most affluent areas were in professional or managerial occupations. By contrast half of the people in the most deprived areas were in semi-routine or routine occupations.

The overall regression model was significant: *R*^2^ = 0.092, s.e. = 3.956, *F*_11, 744_ = 6.729, *p* < 0.001. The regression coefficients shown in table 3 confirm our pre-registered hypothesis that indices of multiple deprivation and socio-economic classification were statistically significant and independent predictors of time preferences. People in the lower supervisory and technical category, and those in the routine and semi-routine category were more impulsive than those in the professional category. Similarly, people in the two most deprived categories were more impulsive than people in the least deprived category. Neither age nor gender predicted time preferences. Age education ended failed to reach the criterion for significance.

**Table 3.** Regression coefficients for the pre-registered analysis.

predictor variable	regression coefficients					*collinearity*	
	*b*	s.e.	*ß*	*t*	*p*	*Tol.*	*VIF*
age	0.001	0.025	0.002	0.044	0.965	0.911	1.098
gender	0.556	0.304	0.067	1.828	0.068	0.915	1.093
age education ended	0.182	0.096	0.081	1.891	0.068	0.678	1.476
*socio-economic status*							
professional versus intermediate	0.669	0.519	0.051	1.289	0.198	0.796	1.256
professional versus small employers	−0.362	0.473	−0.030	−0.765	0.445	0.800	1.250
professional versus lower technical	−2.346	0.570	−0.163	−0.4111	<0.001	0.789	1.268
professional versus routine	−1.151	0.418	−0.124	−2.751	0.006	0.611	1.637
*index of multiple deprivation*							
1 versus 2	−0.049	0.402	0.005	0.121	0.904	0.657	1.522
1 versus 3	0.311	0.443	0.030	0.703	0.482	0.693	1.442
1 versus 4	−1.120	0.481	−0.098	−2.327	0.020	0.699	1.430
1 versus 5	−1.089	0.521	−0.087	−2.092	0.037	0.709	1.410

### 3.2. Extended analyses

The dataset allows further analyses to test our hypothesis and allows us to examine other variables that might confound or contribute to individual differences in decision-making. For example, subjective social status can be a better predictor of health outcomes than objective measures such as socio-economic group [59], and intelligence could be the common underlying factor between occupational group, or education. We were able to compute a proximal measure of cognitive function that approximated to a single measure of executive function (see Material and methods). The resulting model had 986 data points and was significant: *R*^2^ = 0.075, s.e. = 3.970, *F*_9, 984_ = 8.783, *p* < 0.001. The regression coefficients for each predictor variable are shown in table 3. Both cognitive function and subjective social status were predicted time preferences, but these were independent of deprivation and socio-economic status. The regression coefficients are shown in table 4. Gender was also a predictor of time preference in this analysis indicating that men were more impulsive than women.

**Table 4.** Regression coefficients for the extended analysis.

predictor variable	regression coefficients					*collinearity*	
	*b*	s.e.	*ß*	*t*	*p*	*Tol.*	*VIF*
age	0.036	0.022	0.052	1.614	0.107	0.908	1.102
gender	0.569	0.268	0.069	2.123	0.034	0.893	1.120
*socio-economic status*							
professional versus intermediate	0.011	0.426	0.001	0.026	0.979	0.795	1.258
professional versus small employers	−0.595	0.416	−0.048	−1.431	0.153	0.795	1.198
professional versus lower technical	−1.962	0.492	−0.132	−3.991	<0.001	0.861	1.162
professional versus routine	1.011	0.348	−0.110	−2.905	0.004	0.658	1.520
*index of multiple deprivation*							
1 versus 2	0.114	0.346	0.012	0.329	0.743	0.670	1.493
1 versus 3	0.525	0.383	0.050	1.372	0.170	0.707	1.414
1 versus 4	−1.021	0.412	−0.089	−2.480	0.013	0.725	1.380
1 versus 5	−0.821	0.455	−0.065	1.802	0.072	0.721	1.387
cognitive function	0.389	0.145	0.091	2.687	0.007	0.819	1.221
subjective social status	0.023	0.008	0.091	2.723	0.007	0.842	1.187

The pre-registered and extended analyses yielded similar results. In both analyses, the prediction that indices of deprivation predict individual differences in impulsive decision-making was confirmed. In both analyses there were statistically significant differences in decision-making between people from professional occupations and people from both lower supervisory and technical occupations, and semi-routine and routine occupations. However, in the extended analysis the difference in impulsivity between the least deprived group and the most deprived group failed to reach significance once the measure of cognitive function and subjective social status were included. We re-ran the regressions by omitting these two variables separately. When subjective social status was omitted, but not cognitive function, it was observed that the most deprived group was more impulsive than the least deprived group (*b* = −1.041, s.e. = 0.430, ß = −0.084, *t* = −2.421, *p* = 0.016). Neither variable appeared to be related to gender.

## 4. Discussion

Economic scarcity is thought to be associated with individual differences in decision-making [60,61]. We tested this by comparing time preferences for different socio-economic groups, and in geographical areas ranging from the most deprived to the least deprived in England, using the English indices of multiple deprivation. The data supported our hypotheses: people in technical or routine occupations tended to prefer smaller-sooner rewards compared with people in professional or intermediate occupations. Similarly, people in the most deprived areas were more likely to prefer smaller-sooner rewards than people from the least deprived areas. We sought to exclude potentially confounding variables such as educational attainment, cognitive function or subjective social status. To do so, we constructed a variable to measure cognitive function as a proxy for fluid intelligence, and controlled for the length of education. We also included a measure of subjective social status because this can be a better predictor of health than objective measures [62]. Cognitive function and subjective social status predicted time preferences. The data clearly show that people in more deprived geographical areas make more impulsive choices than people in more affluent geographical areas. This finding is independent of our proximal indices of either education or cognitive function. We regard this as strong evidence for the scarcity hypothesis. But there is more than one potential mechanism by which scarcity can affect individual differences in choice behaviour.

### 4.1. Impulsivity as foraging

Decision-making in humans shares many similarities with foraging behaviour in other animals [28] and choice tasks that indicate time preferences are strikingly similar to models of optimal foraging, and a number of researchers have drawn parallels between them [29–31].

In the model described by Shafir and colleagues [5,60,63], the experience of scarcity causes a cognitive shift focused on resources. In this model, decisions appear impulsive because immediate rewards are more salient than later rewards. However, once scarcity is removed there is nothing distinctive about the cognitive processes or choices made by the decision-maker. The removal of scarcity should eliminate impulsive decision-making. On the other hand, the evidence from studies of delay of gratification [37,39] suggests that impulsivity appears in early childhood and may persist across the lifespan.

The basic premise is as follows: the experience of a scarcity or uncertainty in resources causes adaptation of foraging behaviour. This adaptation becomes a stable individual difference that as an adult and in an abundant environment becomes maladaptive, leaving the individual vulnerable to addiction and other impulse control disorders. We propose that the experience of scarce or uncertain resources causes an adaptive shift in choice behaviour that motivates the individual to consume proportionately more of the currently available resources than they would when resources are abundant, in the expectation that future resources will also be unpredictable. The consequence of setting the parameters that govern choice behaviour at an early age leaves the child who experiences scarcity relatively more vulnerable to obesity, addiction and debt as an adult. Individual differences in decision-making may result from the foraging responses that make sense in different environmental conditions such as scarcity of resources. For example, in periods of abundant resources it does not make sense to deplete the available resources, since these could be preserved for the future. Thus, in periods of abundance it is more optimal to exhibit self-control or delay gratification. However, in periods of scarcity it makes more sense to deplete the available resources even if that results in an uncertain future. The classic finding in humans is that children who performed poorly in the original Stanford marshmallow test had poorer life outcomes [37,64] including higher BMI, alcohol and drug use.

In our model, people who encounter periods of scarcity acquire a foraging strategy that becomes a stable individual difference in adulthood choice behaviour. Behavioural evidence from other species is consistent with this hypothesis. For example, developmentally disadvantaged starlings tend to overmatch when foraging and are physically larger in adulthood than developmentally advantaged birds [65]. Similarly, rats show greater sensitivity to resource allocation in predictable environments compared with unpredictable environments [66]. Pigeons show rapid adjustments in habitat matching when resource availability changes and that at group-level sensitivity decreases as the unpredictability of the resource availability increases [67]. Although the data reported here are not able to discriminate between these hypotheses there is considerable evidence that early economic environment can have profound effects on adult health, including increased mortality from causes associated with impulsive choice such as alcohol and tobacco use, in adults who were born into less-affluent social groups [68,69].

### 4.2. Limitations

This research is based on an analysis of secondary data that means we are unable to determine that the association between deprivation and impulsive choice is a causal relationship. Despite being based on a large sample of older adults, only a proportion were selected by the survey team to take part in the risk module, which limits the sample size available to us. The age profile of the sample does not allow us to directly test the causal relationship between early environmental scarcity and individual differences in decision-making. This was further limited by incomplete responses or missing data for some of the variables, particularly age education ended. While the extended analysis is less affected by missing data, this ought to be considered when interpreting the results, as the data is unlikely to be missing completely at random.

## 5. Conclusion

People who experience economic scarcity appear to make more impulsive choice than people who live in more affluent areas. Similarly, people who work in manual occupations tend to be more impulsive than those who work in professional occupations. These effects are additional to, and independent of, the effects of education or cognitive function. We believe that the experience of economic scarcity has a causal influence on time preference that can become a relatively stable individual difference in decision-making, which in turn provides an explanation for the uneven distribution of addictive behaviours across social groups. Future research using life-course longitudinal data would test this theory.

## Data Availability

The data were collected as part of the English Longitudinal Study of Ageing and are freely available from the UK Data Archive https://beta.ukdataservice.ac.uk/datacatalogue/studies/study?id=5050. However, access to some of the data is ‘controlled' by special licence (https://ukdataservice.ac.uk/help/access-policy/types-of-data-access/) because it contains detailed geographies of respondents' locations including postcodes and grid references which may render some individuals to be personally identifiable. The special licence is to ensure that only qualified and responsible researchers have access to those parts of the dataset. These data are open in the sense that they are available to researchers, but only on request from the UK Data Archive. The data are provided in electronic supplementary material [70].
